# Review of the clinical pharmacokinetics of artesunate and its active metabolite dihydroartemisinin following intravenous, intramuscular, oral or rectal administration

**DOI:** 10.1186/1475-2875-10-263

**Published:** 2011-09-13

**Authors:** Carrie A Morris, Stephan Duparc, Isabelle Borghini-Fuhrer, Donald Jung, Chang-Sik Shin, Lawrence Fleckenstein

**Affiliations:** 1 Pharmaceutical Sciences and Experimental Therapeutics, University of Iowa College of Pharmacy, 115 South Grand Avenue, Iowa City, IA 52242, USA; 2 Medicines for Malaria Venture, International Center Cointrin, 20 route de Pré-Bois, 1216 Cointrin, Geneva, Switzerland; 3 Pharmaceutical Research Services, 1300 Seaport Blvd, Suite 500, Redwood City, California 94063, USA; 4 Shin Poong Pharmaceuticals, 748-31, Yoksam-Dong, Kangnam-Ku, Seoul 135-925, Republic of Korea

## Abstract

Artesunate (AS) is a clinically versatile artemisinin derivative utilized for the treatment of mild to severe malaria infection. Given the therapeutic significance of AS and the necessity of appropriate AS dosing, substantial research has been performed investigating the pharmacokinetics of AS and its active metabolite dihydroartemisinin (DHA). In this article, a comprehensive review is presented of AS clinical pharmacokinetics following administration of AS by the intravenous (IV), intramuscular (IM), oral or rectal routes. Intravenous AS is associated with high initial AS concentrations which subsequently decline rapidly, with typical AS half-life estimates of less than 15 minutes. AS clearance and volume estimates average 2 - 3 L/kg/hr and 0.1 - 0.3 L/kg, respectively. DHA concentrations peak within 25 minutes post-dose, and DHA is eliminated with a half-life of 30 - 60 minutes. DHA clearance and volume average between 0.5 - 1.5 L/kg/hr and 0.5 - 1.0 L/kg, respectively. Compared to IV administration, IM administration produces lower peaks, longer half-life values, and higher volumes of distribution for AS, as well as delayed peaks for DHA; other parameters are generally similar due to the high bioavailability, assessed by exposure to DHA, associated with IM AS administration (> 86%). Similarly high bioavailability of DHA (> 80%) is associated with oral administration. Following oral AS, peak AS concentrations (Cmax) are achieved within one hour, and AS is eliminated with a half-life of 20 - 45 minutes. DHA Cmax values are observed within two hours post-dose; DHA half-life values average 0.5 - 1.5 hours. AUC values reported for AS are often substantially lower than those reported for DHA following oral AS administration. Rectal AS administration yields pharmacokinetic results similar to those obtained from oral administration, with the exceptions of delayed AS Cmax and longer AS half-life. Drug interaction studies conducted with oral AS suggest that AS does not appreciably alter the pharmacokinetics of atovaquone/proguanil, chlorproguanil/dapsone, or sulphadoxine/pyrimethamine, and mefloquine and pyronaridine do not alter the pharmacokinetics of DHA. Finally, there is evidence suggesting that the pharmacokinetics of AS and/or DHA following AS administration may be altered by pregnancy and by acute malaria infection, but further investigation would be required to define those alterations precisely.

## Background

Derivatives of the naturally occurring endoperoxide anti-malarial artemisinin form the foundation of the current global treatment approach for *Plasmodium falciparum *malaria. These derivatives, including artesunate (AS), artemether and dihydroartemisinin, produce more profound reductions in parasitaemia and more rapid symptom relief than agents from any other anti-malarial class. Of these derivatives, AS is the most therapeutically versatile agent. As with the other derivatives, AS can be administered orally for the treatment of uncomplicated malaria. Specifically, artemisinin-based combination therapies containing AS partnered with longer acting anti-malarial agents, such as mefloquine or sulphadoxine-pyrimethamine, are extensively utilized for oral treatment of uncomplicated falciparum malaria [1]. Among the artemisinin derivatives, however, only AS displays sufficient water solubility to be administered intravenously; per the World Health Organization treatment guidelines, intravenous AS is the preferred therapy for severe malaria infection in both adult and paediatric patients [1]. AS can also be administered intramuscularly or rectally, with AS suppositories for rectal administration representing a means of initiating treatment of severe malaria before patients are referred to distant facilities for intravenous therapy.

Given the therapeutic significance and versatility of AS, and the necessity of appropriate dosing to avoid suboptimal efficacy or encouragement of resistance, research defining the pharmacokinetics (PK) of AS, and its active metabolite dihydroartemisinin (DHA), is of substantial clinical relevance. The intent of this review is to examine clinical pharmacokinetic findings of AS and DHA following AS administration by the intravenous (IV), intramuscular (IM), oral and rectal routes. To this end, an extensive literature search was conducted utilizing the PubMed database and the bibliographies of identified articles in order to locate AS clinical pharmacokinetic studies in which parameters for AS and/or DHA are reported. The PubMed database was searched using combinations of the following search terms: artesunate, dihydroartemisinin, artemisinin, and pharmacokinetics. Conference abstracts and non-English language articles were not considered for inclusion in the review.

To facilitate comparison of results among various studies, units for these parameters were converted, as necessary, to a uniform scale as noted in the tables included in this review. Additionally, individual PK analyses and population pharmacokinetic (PopPK) analyses are described separately for each route of administration, where applicable, to enable adequate description of the findings from each analysis method.

### Sources of variation introduced by study methodology

Multiple factors complicate comparison and summation of AS/DHA pharmacokinetic findings across multiple studies, including differences in assay sensitivities, sampling schedules, and choice of anticoagulant for blood sample collection. Differences in sampling schedules are of particular importance in comparisons of AS pharmacokinetic parameters; a relative lack of sampling points in the early post-dose period can result in much of subjects' AS exposure being missed. With regard to choice of anticoagulant, if fluoride-oxalate, rather than heparin, is included as the anticoagulant in blood sample collection tubes, *ex vivo *plasma esterase degradation of AS to DHA is greatly inhibited [2]. This inhibition allows for greater preservation of a subject's AS concentrations at the time of blood sample collection. However, fluoride-oxalate may also result in greater erythrocyte shrinkage than heparin, and therefore increased plasma volume [2]. Given these sources of variation, differences in pharmacokinetic findings among the studies described in this review cannot necessarily be regarded as solely related to whatever specific demographic or clinical features characterize the study subjects.

### Artesunate and DHA protein binding

Binding of AS to human plasma proteins has been investigated utilizing equilibrium dialysis with [^14^C] artesunate. AS was determined to be 75% protein bound at plasma concentrations less than 125 ng/mL, and 62% protein bound at higher concentrations [3]. DHA plasma protein binding, when measured by similar means, was determined to be 82% at plasma concentrations less than 25 ng/mL and 66% at higher concentrations [4]. DHA percent bound was also assessed by ultrafiltration in patients with malaria infection (falciparum or vivax), Vietnamese healthy volunteers, and Caucasian volunteers and determined to be 93%, 88%, and 91%, respectively [5]. However, as AS and DHA are both high extraction ratio drugs [6], any alterations in patients' protein binding capacity would not be expected to produce clinically relevant changes in the clearance of either agent.

### Intravenous administration: artesunate pharmacokinetics

The pharmacokinetic results of the identified studies in which intravenous AS PK were assessed are presented in Tables 1 and 2[7-15]; both AS and DHA PK were described in eight of these studies, with only DHA PK described in the ninth [15]. In clinical settings, IV AS is administered as a bolus injection [16]. As the identified studies replicated this administration method, very high AS maximum concentrations (Cmax) were observed. AS is metabolized through esterase-catalyzed hydrolysis to yield its active metabolite, DHA [17]; this conversion occurs quickly following IV AS administration as indicated by the rapid decline in AS concentrations in the early post-dose period. In six of the eight studies in which AS PK were assessed, average AS half-life following IV AS was determined to be less than five minutes in at least one study cohort. In all of the studies, average AS half-life was determined to be less than fifteen minutes. Finally, per the findings of Li *et al *[11], AS was determined to display dose linearity following IV administration across a dosage range of 0.5 - 8 mg/kg.

**Table 1:** Summary of AS pharmacokinetic results following IV AS administration.

**Ref**.	Subjects & Regimen	Cmax (ng/mL)	Clearance (L/kg/hr)	Volume (L/kg)	Half-life (min)	AUC (ng*hr/mL)
[7] Batty, Le *et al*, 1998	12 Vietnamese adults with vivax malaria 120 mg IV AS over 2 min	13685†^a^	3.01	0.16	2.19	876†

[9] Binh *et al*, 2001	17 healthy Vietnamese volunteers; subjects randomized into two groups, both receiving: 120 mg IV AS over 2 min		3.0; 2.2	0.19; 0.16	2.6; 3.3	846; 1269†

[10] Ilett *et al*, 2002	23 Vietnamese adults with uncomplicated falciparum malaria; subjects randomized into two groups, both receiving: 120 mg IV AS over 2 min	16146; 16530†	2.8; 2.1	0.22	3.2	1038; 1230†

[11] Li *et al*, 2009	30 healthy volunteers 0.5, 1, 2, 4, or 8 mg/kg IV AS over 2 min	4797; 6128; 19420; 36100; 83340	1.3; 18; 1.3; 1.4; 1.6†	Vss: 0.092; 0.187; 0.106; 0.109; 0.165†	7.2; 8.4; 14.4; 9.0; 12.6†	386; 593; 1595; 3038; 6994†

[12] Nealon *et al*, 2002	28 paediatric Gabonese patients with severe malaria randomized into two groups Group 1: 2.4 mg/kg IV AS Group 2: 1.2 mg/kg IV AS	Group 1: 29677^a^ Group 2: 15369^a^	Group 1: 3.12†^a^ Group 2: 4.26†^a^	Group 1 Vss: 0.17†^a^ Group 2 Vss: 0.44†^a^	Group 1: 1.5^a^ Group 2:11.5^a^	Group 1: 1042†^a^ Group 2: 555†^a^

[13] Newton *et al*, 2006	17 adults with severe falciparum malaria in Thailand 2.4 mg/kg IV AS over 2 min [2.1 (1.4 - 2.8 mg/kg)]	130^+^†^a^	64^a^	Vss: 15.2^a^	13.2^+^†^a^	49.2†^a^

[8] Batty. Thu *et al*, 1998	26 adult uncomplicated falciparum malaria patients in Vietnam 120 mg IV AS over 2 min		2.33^a^	0.140^a^	2.73	1146†

[14] Davis *et al*, 2001	30 parasitaemic adults with falciparum malaria Group 1: 12 with complications (120 mg IV AS over 2 min) Group 2: 8 without complications (120 mg IV AS over 2 min) Group 3: 10 with moderately severe complications (240 mg IV AS infused over 4 hours; samples taken after infusion discontinued)		Group 1: 1.63 Group 2: 2.49 Group 3: 3.07	Group 1: 0.08 Group 2: 0.24 Group 3: 0.23	Group 1: 2.3 Group 2: 4.3 Group 3: 3.2	

Values given as mean unless otherwise specified.

†Units converted to uniform scale +The authors note that Cmax is likely underestimated and half-life overestimated due to the lack of usable data from six patients with extremely rapid AS elimination. a.Median

**Table 2:** Summary of DHA pharmacokinetic results following IV AS administration.

**Ref**.	Subjects & Regimen	Cmax (ng/mL)	Tmax (min)	Clearance (L/kg/hr)	Volume (L/kg)	Half-life (min)	AUC (ng*hr/mL)
[7] Batty, Le *et al*, 1998	12 Vietnamese adults with vivax malaria 120 mg IV AS over 2 min	2192†^a^	8^a^	1.10	0.92	36.7	1845†

[9] Binh *et al*, 2001	17 healthy Vietnamese volunteers; subjects randomized into two groups, both receiving: 120 mg IV AS over 2 min	1507; 1678†	9; 16			53; 47	

[10] Ilett *et al*, 2002	23 Vietnamese adults with uncomplicated falciparum malaria; subjects randomized into two groups, both receiving: 120 mg IV AS over 2 min	2758; 2730†^a^	7; 9^a^	0.64; 0.48	0.8; 0.55	59; 50	2872; 3298†

[11] Li *et al*, 2009	30 healthy volunteers 0.5, 1, 2, 4, or 8 mg/kg IV AS over 2 min	428; 802; 1286; 3148; 4744	9.6; 15; 9.6; 7.2; 24†	1.3; 0.98; 1.1; 0.86; 0.82†	Vss: 1.734; 2.201; 1.860; 1.701; 2.403†	57.6; 92.4; 69.0; 82.2; 128.4†	385; 1082; 1850; 4886; 10410

[12] Nealon *et al*, 2002	28 paediatric Gabonese patients with severe malaria randomized into two groups Group 1: 2.4 mg/kg IV AS Group 2: 1.2 mg/kg IV AS	Group 1: 3011†^a^ Group 2: 1584†^a^	Group 1: 0.5^a^ Group 2: 1.4^a^	Group 1: 2.16†^a^ Group 2: 1.08†^a^	Group 1 Vss: 0.75^a^ Group 2 Vss: 0.77^a^	Group 1: 20.7^a^ Group 2: 32.0^a^	Group 1: 923†^a^ Group 2: 737†^a^

[13] Newton *et al*, 2006	17 adults with severe falciparum malaria in Thailand 2.4 mg/kg IV AS over 2 min [2.1 (1.4 - 2.8 mg/kg)]	605†^a^	Tmax reached by 15 min	5.6^a^	Vss: 1.9^a^	20.4†^a^	418†^a^

[8] Batty. Thu *et al*, 1998	26 adult uncomplicated falciparum malaria patients in Vietnam 120 mg IV AS over 2 min	2648†^a^	9.0^a^	0.75^a^	0.76	40.2	2377†

[14] Davis *et al*, 2001	30 parasitaemic adults with falciparum malaria Group 1: 12 with complications Group 2: 8 without complications Group 1 & 2: 120 mg IV AS over 2 min Group 3: 10 with moderately severe complications (240 mg IV AS infused over 4 hours; samples taken after infusion discontinued)	Group 1: 2417† Group 2: 2531† Group 3: 910 †	Group 1: 10.4 Group 2: 9.9 Group 3: 240 (end of infusion)	Group 1: 1.09 Group 2: 0.73 Group 3: 0.73	Group 1: 0.77 Group 2: 1.01 Group 3: 0.78	Group 1: 40.0 Group 2: 64.1 Group 3: 46.2	Group 1: 2078† Group 2: 2559† Group 3: 5573†

Krishna *et al*, 2001 [15]	34 Ghanaian children (8 months - 7 years) with moderate falciparum malaria Group 1 & 2: AS rectal suppository, IV AS 2.4 mg/kg 12 hr later Group 3: 2.4 mg/kg IV AS, AS rectal suppository 12 hr later	Groups 1 & 2: 1280†^a^ Group 3: 1592 †^a^	Groups 1 & 2: 12†^a^ Group 3: 12†	Groups 1 & 2: 1.5^a^ Group 3: 1.0^a^	Groups 1 & 2: 0.6^a^ Group 3: 0.9^a^	Groups 1 & 2: 18†^a^ Group 3: 31.8†^a^	Groups 1 & 2: 1166†^a^ Group 3: 1706†^a^

Values given as mean unless otherwise specified.

†Units converted to uniform scale a. Median

Examination of the AS clearance and volume estimates summarized in Table 1 indicates that the parameters obtained by Newton *et al *[13] are dissimilar from the parameters obtained in other studies, likely due to lack of sampling prior to 15 minutes post-dose; the other summarized studies included a sampling point at or before five minutes. The remaining seven studies have more consistent clearance and volume estimates, with averages ranging from 1.3 - 4.26 L/kg/hr and 0.08 - 0.44 L/kg, respectively. A majority of the estimates range between 2 - 3 L/kg/hr for clearance and 0.1 - 0.3 L/kg for volume.

### Intravenous administration: DHA pharmacokinetics

In all of identified IV AS studies, the time to maximum concentration (Tmax) of DHA following IV AS administration was less than 25 minutes. DHA metabolism occurs through conjugation of DHA by the UDP-glucuronosyltransferase system, with UGT1A9 and UGT2B7 being the primary responsible isoforms [18]. This DHA elimination process occurs somewhat more slowly than the esterase-catalyzed AS elimination, with average half-life estimates for DHA following IV AS administration ranging from 18 minutes to 2.14 hours, with eight of the nine studies citing average estimates for all study cohorts of less than 65 minutes and the majority of estimates falling between 30 - 60 minutes. DHA apparent clearance (Cl/F) and volume of distribution (V/F) averages ranged from 0.48 - 5.6 L/kg/hr and 0.55 - 2.403 L/kg, with a majority of the estimates averaging 0.5 - 1.5 L/kg/hr for clearance and 0.5 - 1.0 L/kg for volume. As with AS, DHA displayed dose-linearity across an IV AS dosage range of 0.5 - 8 mg/kg [11].

### Intravenous administration: bioassay results

The anti-malarial bioassay method for determination of AS/DHA plasma concentrations provides values in DHA equivalents reflecting the contribution of both AS and DHA present in the sample. An assessment of anti-malarial bioactivity in patients with acute uncomplicated falciparum malaria administered 2 mg/kg IV AS yielded estimates for half-life, volume of distribution at steady state (Vss), and clearance of 0.73 hours, 0.61 L/kg, and 0.83 L/kg/hr, respectively [19]. Bioassay data were also used to compute the pharmacokinetic parameters following administration of 2.4 mg/kg IV AS to thalassaemic and healthy non-thalassaemic adults. The reported half-life estimates for normal and thalassaemic subjects were 1.37 and 1.95 hours, respectively, with Tmax for bioassay activity occurring by the first sampling point (15 minutes) [20]. As would be expected given the more extended half-life of DHA, these bioassay results appear somewhat more reflective of the DHA results than the AS results derived from traditional analytical methods.

### Intramuscular administration: artesunate and DHA pharmacokinetics

Tables 3 and 4[10,12,21] summarize the results of the identified studies examining AS and DHA PK following IM AS administration. Average estimates of the absolute bioavailability of IM AS, as determined by DHA concentrations, were 86.4% [12] and 88% [10] in adult and paediatric falciparum malaria patients, respectively. The average time to maximum AS concentrations following IM administration ranged from 7.2 - 12 minutes; average AS half-life ranged from 25.2 - 48.2 minutes. This more extended AS half-life following IM administration of AS presumably indicates that the AS elimination rate was being limited by the rate of absorption from the site of injection. Average estimates for AS apparent clearance and volume of distribution ranged from 2.4 - 3.48 L/kg/hr and 1.09 - 3.98 L/kg, respectively. As would be expected given the high bioavailability of IM AS, these estimates for apparent clearance are not strikingly higher than those obtained following IV AS administration.

**Table 3:** Summary of AS pharmacokinetic results following IM AS administration.

**Ref**.	Subjects & Regimen	Cmax (ng/mL)	Tmax (min)	Clearance (L/kg/hr)	Volume (L/kg)	Half-life (min)	AUC (ng*hr/mL)
[10] Ilett *et al*, 2002	11 Vietnamese adults with uncomplicated falciparum malaria 120 mg AS administered by intramuscular injection	884†^a^	12^a^	2.9	2.6	41	999†

[21] Hien *et al*, 2004	9 Vietnamese adults with severe falciparum malaria 2.4 mg/kg AS administered by intramuscular injection	2195†^a^		2.84^a^	1.09^a^	30^a^	856†^a^

[12] Nealon *et al*, 2002	28 paediatric Gabonese patients with severe malaria randomized into two groups Group 1: 2.4 mg/kg IV AS, followed by 1.2 mg/kg IM AS 12 hours later Group 2: 2.4 mg/kg IM AS, followed by 1.2 mg/kg IV AS 12 hours later	Group 1: 615†^a^ Group 2: 661†^a^	Group 1: 7.2^a^ Group 2: 8.0^a^	Group 1: 2.4†^a^ Group 2: 3.48†^a^	Group 1 Vss/F: 2.07^a^ Group 2 Vss/F: 3.98^a^	Group 1: 25.2^a^ Group 2: 48.2^a^	Group 1: 535†^a^ Group 2: 544†^a^

Values given as mean unless otherwise specified.

†Units converted to uniform scale a. Median

**Table 4:** Summary of DHA pharmacokinetic results following IM AS administration.

**Ref**.	Subjects & Regimen	Cmax (ng/mL)	Tmax (min)	Clearance (L/kg/hr)	Volume (L/kg)	Half-life (min)	AUC (ng*hr/mL)
[10] Ilett *et al*, 2002	11 Vietnamese adults with uncomplicated falciparum malaria 120 mg AS administered by intramuscular injection	1166†^a^	45^a^	0.73	1.1	64	2474† F: 88%

[21] Hien *et al*, 2004	9 Vietnamese adults with severe falciparum malaria 2.4 mg/kg AS administered by intramuscular injection	870†^a^	35^a^	1.18^a^	1.79^a^	52.7^a^	1496†^a^

[12] Nealon *et al*, 2002	28 paediatric Gabonese patients with severe malaria randomized into two groups Group 1: 2.4 mg/kg IV AS, followed by 1.2 mg/kg IM AS 12 hours later Group 2: 2.4 mg/kg IM AS, followed by 1.2 mg/kg IV AS 12 hours later	Group 1: 341^a^ Group 2: 626^a^	Group 1: 25.9^a^ Group 2: 40.5^a^	Group 1: 2.16†^a^ Group 2: 1.5†^a^	Group 1: Vc/F: 1.2^a^ Vss/F: 1.32^a^ Group 2: Vc/F: 1.2^a^ Vss/F: 1.28^a^	Group 1: 31.9^a^ Group 2: 40.2^a^	Group 1: 396†^a^ Group 2: 1123†^a^ Combined group F: 86.37%^a^

Values given as mean unless otherwise specified.

†Units converted to uniform scale a. Median

Maximum DHA concentrations following IM AS administration occurred, on average, within the first 45 minutes post-dose. Average estimates for DHA half-life, apparent clearance, and apparent volume of distribution following IM AS ranged from 31.9 - 64 minutes, 0.73 - 2.16 L/kg/hr, and 1.1 - 1.7 L/kg, respectively; all of these values are quite similar to those obtained following IV AS administration.

### Oral administration: artesunate pharmacokinetics

Based upon complete metabolism to DHA, AS displays high oral bioavailability when assessed by exposure to its active metabolite DHA. Following intravenous and oral AS, the oral bioavailability of DHA was determined to be 82% in healthy adults [9], 85% in adults with uncomplicated falciparum malaria [8], and 80% in adults with vivax malaria [7]. It should be noted, however, that these bioavailability results may reflect both the absorption of AS, with subsequent conversion to DHA through first-pass or systemic metabolism, as well as direct absorption of DHA following its formation in the gut through acid-dependent chemical hydrolysis [22]. Although physiologically plausible, the extent of such chemical hydrolysis has not been well quantified. Following oral administration, AS concentrations are detectable early, often within 15 minutes post-dose. Peak AS concentrations also occur early, with AS Tmax typically being detected within the first hour post-dose (Table 5; [6,23-37]). These findings suggest that AS is absorbed quickly and without appreciable lag.

**Table 5:** Artesunate Tmax values obtained following oral artesunate administration.

**Ref**.	Subjects	Regimen	Tmax (hours)
[23]	15 healthy Cambodian male adults	4 mg/kg AS once with mefloquine	0.75^a^

[6]	8 healthy adults in Australia	150 mg AS once	0.65 (n = 6)^a^

[24]	20 healthy adult males in Australia	200 mg/day × 3 days alone (Period 1); repeated with mefloquine after washout (Period 2)	Period 1/Day 1: 0.6^b^ Period 1/Day 3: 0.6^b^ Period 2/Day1: 0.5^b^ Period 2/Day 3: 0.6^b^

[25]	6 healthy adults in Geneva	200 mg AS once	0.25 (5/6 subjects) 0.5 (1/6 subjects)

[26]	23 healthy Malaysian adults	200 mg AS once with amodiaquine as fixed or non-fixed product	Fixed:0.26 Non-fixed:0.53

[27]	13 healthy adults in Africa	Mean dose: 4.26 mg/kg with (ACT) or without (AS only) amodiaquine as single dose	AS only: 0.62 ACT: 0.86

[28]	8 healthy male Thai adults	300 mg AS (Guilin or Arenco formulation)	Guilin: 0.25^a^ Arenco: 0.31^a^

[29]	10 healthy male Vietnamese adults	200 mg AS once daily × 5 days	Day 1: 0.8^a^ Day 5: 0.8^a^

[30]	12 healthy male Malaysian adults	200 mg AS once	0.66 ± 0.34

[31]	11 male Thai adults with uncomplicated falciparum malaria	200 mg AS once, followed by 100 mg 12 hours later, then 100 mg once daily for another 4 days	Acute: 0.5^a^ Convalescence:1.0^a^

[32]	43 adults with uncomplicated falciparum malaria in Thailand	AS+mefloquine as fixed (200 mg AS) or nonfixed (4 mg/kg AS) combination	Fixed:0.833 (n = 19) Nonfixed:0.925 (n = 23)

[33]	13 male and female adult patients in the DRC with acute uncomplicated falciparum malaria	200 mg AS once daily × 3 days with amodiaquine	1.4 (n = 10)

[34]	86 acute uncomplicated falciparum malaria patients from Malawi and Gambia	1, 2, or 4 mg/kg AS with chlorproguanil and dapsone once daily × 3 days	1 mg/kg: 1.08^a^ 2 mg/kg: 0.55^a^ 4 mg/kg: 1.03^a^

[35]	6 male Thai adults with uncomplicated falciparum malaria and 6 healthy male adults	100 mg AS once	Healthy: 0.71 Patients: not determined

[36]	57 children (2-14 years) with uncomplicated falciparum malaria in Gabon	AS dose (mg/kg/day) with pyronaridine Group A:2.1(1.4-2.4) Group B:3.3(2.4 - 3.9) Group C:4.8(3.0 - 6.1) Group D: 3.8(3.0-4.3)	Group A:0.6 Group B:0.7 Group C:1.0 Group D:0.5

[37]	40 children and adults with uncomplicated falciparum malaria in Pailin, Cambodia and 40 adults with uncomplicated falciparum malaria in Wang Pha, Thailand	At each site: Group 1: AS monotherapy: 2 mg/kg/day × 7 days Group 2: AS 4 mg/kg/day × 3 days + mefloquine	Thailand: Group 1: 0.38^a^ Group 2: 0.50^a^ Cambodia: Group 1:0.50^a^ Group 2: 1.00^a^

Values given as mean unless otherwise specified.

a. Median b. Geometric mean

AS half-life estimates available in the literature are summarized in Table 6 ([28-31,35-37]), with average AS half-life reported for any cohort in the identified studies ranging from 0.36 - 1.2 hours. All studies in which AS half-life was determined cite AS half-life values between 25 and 40 minutes for at least one study cohort. There are few published estimates of AS apparent clearance and volume of distribution assessed following oral AS administration. Teja-Isavadharm *et al *[35] determined mean AS CL/F and V/F to be 20.6 L/kg/hr and 14.8 L/kg, respectively, in six healthy adult subjects. Karbwang *et al *[31] determined average AS CL/F to be 19.2 L/kg/hr in 11 Thai adults during the acute phase of uncomplicated malaria infection and 9.6 L/kg/hr during the convalescent phase. Median AS V/F was 6.8 L/kg during both phases of infection. Finally, in paediatric Gabonese patients with acute falciparum malaria, estimates for average AS CL/F and V/F were 25 - 30 L/kg/hr and 25 - 41 L/kg, respectively [36].

**Table 6:** Artesunate half-life values following oral artesunate administration.

**Ref**.	Subjects	Artesunate regimen	Artesunate half-life (hours)
[29]	10 healthy male Vietnamese adults	200 mg once daily × 5 days	Day 1: 0.43^a^ Day 5: 0.50^a^

[30]	12 healthy male Malaysian adults	200 mg single dose	0.49

[28]	8 healthy male Thai adults	300 mg AS (Guilin or Arenco formulation)	Guilin:0.53 Arenco:0.57

[26]	23 healthy Malaysian adults	200 mg AS + amodiaquine as fixed or non-fixed product	Fixed:0.63 Non-fixed:0.76

[34]	86 acute uncomplicated falciparum malaria patients from Malawi and Gambia	1, 2, or 4 mg/kg AS + chlorproguanil and dapsone once daily × 3 days	1 mg/kg: 0.515^b^ 2 mg/kg: 0.478^b^ 4 mg/kg: 0.467^b^

[31]	11 male Thai adults with uncomplicated falciparum malaria	200 mg AS once, followed by 100 mg 12 hours later, then 100 mg once daily × 4 days	Acute: 0.36^a^ Convalescence: 0.54^a^

[35]	6 male Thai adults with uncomplicated falciparum malaria and 6 healthy male adults	100 mg AS once	Healthy: 0.41 Patients: not determined

[36]	57 children (2-14 years) with uncomplicated falciparum malaria in Gabon	AS dose (mg/kg) with pyronaridine Group A:2.1(1.4-2.4) Group B:3.3(2.4 - 3.9) Group C:4.8(3.0 - 6.1) Group D: 3.8(3.0-4.3)	Group A: 0.8 (n = 12) Group B:1.1 (n = 12) Group C:0.5 (n = 10) Group D:1.2 (n = 13)

[37]	40 children and adults with uncomplicated falciparum malaria in Pailin, Cambodia and 40 adults with uncomplicated falciparum malaria in Wang Pha, Thailand	At each site: Group 1: AS monotherapy: 2 mg/kg/day × 7 days Group 2: AS 4 mg/kg/day × 3 days + mefloquine	Thailand: Group 1: 0.37^a^ Group 2: 0.58^a^ Cambodia: Group 1: 0.29^a^ Group 2: 0.29^a^

Value given as mean unless otherwise specified.

a. Median b. Geometric mean

Given that AS is a high extraction ratio drug, the substantial difference in magnitude of these AS apparent clearance and volume estimates for oral administration, as compared to IV or IM administration, most likely reflects the low bioavailability of AS due to the extensive conversion of AS to DHA during first-pass metabolism.

### Oral administration: DHA pharmacokinetics

For studies defining both AS and DHA PK following oral AS administration, DHA Cmax exceeds AS Cmax, and DHA AUC exceeds AS AUC. Literature results exemplifying this relationship between AS and DHA pharmacokinetic exposure are summarized in Table 7[6,26-30,32,34,35,37-39]. In many of these studies, DHA AUC exceeds AS AUC by more than 10-fold, when considered on either a nmol*hr/mL or ng*hr/mL basis. It is in part due to this disparity in exposure that AS is often considered essentially a pro-drug for DHA following oral AS administration. The maximum concentration for DHA typically occurs within two hours post-dose. DHA is eliminated more slowly than AS following oral AS administration. DHA half-life was determined to be longer than AS half-life for all studies in which both parameters were assessed. For the studies in which DHA half-life was estimable (Table 8; [7-9,23-32,34-43]), the average DHA half-life ranged from 0.49 hours to 3.08 hours, with almost half of the half-life estimates being less than one hour and most of the remaining estimates being between 1-2 hours. Overall, most of the half-life estimates fell between 0.5 - 1.5 hours.

**Table 7:** Artesunate and DHA AUC and Cmax values following oral artesunate administration.

**Ref**.	Subjects	Oral AS regimen	AUC (ng*hr/mL)	Cmax (ng/mL)
[29]	10 healthy male Vietnamese adults	200 mg once daily × 5 days	AS^a^ Day 1: 67 Day 5: 60	AS^a^ Day 1:67 Day 5: 58

			DHA^a^ Day 1:1158 Day 5:1300	DHA Pooled:654

[30]	12 healthy male Malaysian adults	200 mg AS once	AS 119	AS 256.3

			DHA AUC_0-t_:1331	DHA 873.7

[27]	13 healthy adults in Africa	Mean dose: 4.26 mg/kg with (ACT) or without (AS only) amodiaquine single dose	AS AS only: 206.4 ACT: 183.3	AS AS only: 231.8 ACT: 141.6

			DHA AS only: 2044.4 ACT: 1410.5	DHA AS only: 844.5 ACT: 446.2

[28]	8 healthy male Thai adults	300 mg AS (Guilin and Arenco formulations)	AS Guilin: 406 Arenco: 190.8	AS Guilin: 397 Arenco: 194

			DHA Guilin: 1630 Arenco: 2600	DHA Guilin: 500 Arenco:928

[6]	8 healthy adults in Australia	150 mg once	AS AUC_0-6 hr_:154 (n = 6)	AS^a^ 111 (n = 6)

			DHA AUC_0-6 hr_: 824	DHA^a^ 546

[26]	23 healthy Malaysian adults	200 mg once with amodiaquine as fixed or non-fixed product	AS† Fixed: 391.1 Non-fixed: 213.2	AS† Fixed: 333 Non-fixed: 444

			DHA† Fixed: 1468.9 Non-fixed: 1656.0	DHA† Fixed: 609.8 Non-fixed:874.5

[34]	86 acute uncomplicated falciparum malaria patients from Malawi and Gambia	1, 2, or 4 mg/kg AS with chlorproguanil and dapsone once daily × 3 days	AS^b^ 1 mg/kg: 64.6 (n = 16) 2 mg/kg:151(n = 19) 4 mg/kg: 400(n = 23)	AS^b^ 1 mg/kg: 48.9 2 mg/kg: 106 4 mg/kg: 224

			DHA^b^ 1 mg/kg: 538 (n = 24) 2 mg/kg: 1445(n = 29) 4 mg/kg: 383(n = 23)	DHA^b^ 1 mg/kg: 228 2 mg/kg: 581 4 mg/kg: 1414

[39]	21 children (5 - 13 years) with uncomplicated malaria in Uganda	4 mg/kg once daily with amodiaquine × 3 days	AS 113 (data pooled from all subjects)	AS 51 (data pooled from all subjects)

			DHA^a^ 1404	DHA^a^ 473

[32]	43 adults with uncomplicated falciparum malaria in Thailand	200 mg/day for fixed dose AS-mefloquine tablet (n = 20) or 4 mg/kg/day as nonfixed (n = 23) AS-mefloquine	AS AUC_0-t_: Fixed:310 (n = 19) Nonfixed: 419 (n = 21)	AS Fixed:255 (n = 19) Nonfixed:451 (n = 23)

			DHA AUC_0-t_: Fixed:3027 Nonfixed: 3633	DHA Fixed:1234 Nonfixed:2043

[35]	6 male Thai adults with uncomplicated falciparum malaria and 6 healthy male adults	100 mg AS once	AS AUC_0-12 hr_ Healthy: 97 Patients: not determined	AS Healthy: 114 Patients: not determined

			DHA AUC_0-12 hr_ Healthy: 501 Patients: 1144	DHA Healthy: 339 Patients: 554

[36]	57 children (2-14 years) with uncomplicated falciparum malaria in Gabon	AS dose (mg/kg) Group A:2.1(1.4-2.4) Group B:3.3(2.4 - 3.9) Group C:4.8(3.0 - 6.1) Group D: 3.8(3.0-4.3)	AS Group A: 104 (n = 12) Group B: 154 (n = 12) Group C:232 (n = 10) Group D: 179 (n = 13)	AS Group A: 93 Group B:154 Group C: 287 Group D: 171

		Administered with pyronaridine	DHA Group A: 1055 Group B: 1989 Group C: 2961 Group D: 2245	DHA Group A:479 Group B: 940 Group C:1186 Group D:792

[37]	40 children and adults with uncomplicated falciparum malaria in Pailin, Cambodia and 40 adults with uncomplicated falciparum malaria in Wang Pha, Thailand	At each site: Group 1: AS monotherapy: 2 mg/kg/day × 7 days Group 2: AS 4 mg/kg/day × 3 days + mefloquine	AS†^a^ AUC_0-24 hr_ Thailand: Group 1: 128 Group 2: 237 Cambodia: Group 1: 173 Group 2: 338	AS†^a^ Thailand: Group 1: 171 Group 2: 200 Cambodia: Group 1: 270 Group 2: 316

			DHA†^a^ AUC_0-24 hr_ Thailand: Group 1: 1308 Group 2: 2957 Cambodia: Group 1: 1382 Group 2: 4123	DHA†^a^ Thailand: Group 1: 859 Group 2: 1191 Cambodia: Group 1: 802 Group 2: 1590

Values given as mean unless otherwise specified.

†Units converted to uniform scale a. Median b. Geometric mean

**Table 8:** DHA half-life values obtained following artesunate administration.

**Ref**.	Subjects	Artesunate regimen	DHA half-life (hours)
[29]	10 healthy male Vietnamese adults	200 mg once daily × 5 days	0.87 (from pooled data)

[30]	12 healthy male Malaysian adults	200 mg single dose	0.49

[40]	20 male and female healthy Thai adults	4 mg/kg once	0.74^a^

[27]	13 healthy adults in Africa	Mean dose: 4.26 mg/kg with (ACT) or without (AS only) amodiaquine single dose	AS only:1.46 ACT: 3.08

[28]	8 healthy male Thai adults	300 mg AS (Guilin or Arenco formulation)	Guilin: 1.77 Arenco:1.73

[41]	10 healthy Vietnamese males	100 mg AS once	Half-life: 0.55^b^

[25]	6 healthy adults in Geneva	200 mg AS once	0.65

[26]	23 healthy Malaysian adults	200 mg once with amodiaquine as fixed or non-fixed product	Fixed:1.68 Non-fixed:1.42

[24]	20 healthy adult males in Australia	200 mg/day × 3 days alone (Period 1); repeated with mefloquine after washout (Period 2)	Period 1/Day 1: 1.14 Period 1/Day 3: 1.14 Period 2/Day 1: 1.02 Period 2/Day 3: 1.09

[23]	15 healthy Cambodian male adults	4 mg/kg once with mefloquine	1.30^b^

[42]	12 healthy adults	200 mg once	0.68

[34]	86 acute uncomplicated falciparum malaria patients from Malawi and Gambia	1, 2, or 4 mg/kg AS with chlorproguanil and dapsone once daily × 3 days	1 mg/kg: 0.779^b^ 2 mg/kg: 0.917^b^ 4 mg/kg: 1.09^b^

[7]	12 Vietnamese adult male vivax malaria patients	100 mg single dose	0.67 (n = 11)

[8]	26 Vietnamese adult patients with uncomplicated falciparum malaria	100 mg single dose	0.66 (n = 16)

[39]	21 children (5 - 13 years) with uncomplicated malaria in Uganda	4 mg/kg once daily with amodiaquine × 3 days	1.3^a^

[38]	24 children with uncomplicated falciparum malaria in Gabon	4 mg/kg once daily for 3 days in one of two formulations (blister pack and fixed dose) of AS/mefloquine	Fixed dose: 0.9(n = 9)^a^ Blister pack: 1.0 (n = 11)^a^

[32]	43 adults with uncomplicated falciparum malaria in Thailand	200 mg/day for fixed dose AS-mefloquine tablet or 4 mg/kg/day as nonfixed AS-mefloquine	Fixed: 1.1 (n = 14) Nonfixed: 0.8 (n = 18)

[31]	11 male Thai adults with uncomplicated falciparum malaria	200 mg AS once, followed by 100 mg 12 hours later, then 100 mg once daily × 4 days	Acute: 0.64^a^ Convalescence: 0.66^a^

[43]	24 pregnant Karen women in the 2^nd ^and 3^rd ^trimesters with uncomplicated falciparum malaria	4 mg/kg once daily × 3 days with atovaquone plus proguanil	Half-life: 1.0 (n = 13)^a^ PopPK half-life estimate: 1.81 hr

[35]	6 male Thai adults with uncomplicated falciparum malaria and 6 healthy male adults	100 mg AS once	Healthy: 0.85 Patients: 1.06

[9]	8 Vietnamese adults with uncomplicated falciparum malaria and 10 healthy Vietnamese adults	150 mg once	Healthy: 0.77 Patients: 0.88

[53]	26 2^nd ^and 3^rd ^trimester pregnant women with asymptomatic falciparum parasitaemia, the same women postpartum, and 25 non-pregnant asymptomatic, parasitaemic controls	200 mg once	Pregnant: 1.28^a^ Postpartum:1.63^a^ Controls: 1.41^a^

[36]	57 children (2-14 years) with uncomplicated falciparum malaria in Gabon	AS dose (mg/kg) Group A:2.1(1.4-2.4) Group B:3.3(2.4 - 3.9) Group C:4.8(3.0 - 6.1) Group D: 3.8(3.0-4.3) Administered with pyronaridine	Group A:1.0 Group B: 0.9 Group C: 1.2 Group D:1.2

[37]	40 children and adults with uncomplicated falciparum malaria in Pailin, Cambodia and 40 adults with uncomplicated falciparum malaria in Wang Pha, Thailand	At each site: Group 1: AS monotherapy: 2 mg/kg/day × 7 days Group 2: AS 4 mg/kg/day × 3 days + mefloquine	Thailand: Group 1: 0.71^a^ Group 2: 0.85^a^ Cambodia: Group 1: 0.84^a^ Group 2: 0.77^a^

Estimates from PopPK studies are described in the text. Values given as mean unless otherwise specified.

a. Median b. Geometric mean

DHA CL/F and V/F estimates obtained following oral AS administration are limited. Teja-Isavadharm *et al *[35] determined DHA CL/F to be 3.35 L/kg/hr in six healthy adults and 1.01 L/kg/hr in six parasitaemic adults. DHA apparent volume of distribution values were 4.14 L/kg and 1.55 L/kg in healthy subjects and malaria patients, respectively. In Gabonese children with malaria, average DHA CL/F and V/F averaged 2.3 - 2.7 L/kg/hr and 1.6 - 4.2 L/kg, respectively [36]. Orrell *et al *[27], Davis *et al *[24], and Zhang *et al *[41] computed DHA CL/F, but did not provide values adjusted for body weight. Adjusting CL/F using the average body weight in these studies yields apparent clearance estimates of 2.2 L/kg/hr (AS alone) and 2.7 L/kg/hr (with amodiaquine) for Orrell *et al *[27], 1.4 - 1.7 L/kg/hr for Davis *et al *[24], and 1.8 L/kg/hr for Zhang *et al *[41]. Adjusted DHA V/F values ranged from 1.6 - 2.6 L/kg [24].

### Oral administration: bioassay results

As was previously observed for IV bioassay studies, PK parameters derived from bioassay data obtained following oral AS administration appear to more closely resemble DHA rather than AS parameters. For example, in the four identified studies including bioassay data following oral AS administration, the average Tmax for bioactivity ranged from 0.75 - 1.7 hours and average half-life from 0.71 - 1.17 hours [19,44-46].

### Oral administration: population pharmacokinetic analyses

Four population pharmacokinetic analyses describing AS and/or DHA PK following oral AS administration were identified, including two conducted with data from pregnant women (described under *Artesunate and DHA pharmacokinetics in pregnant women*, below), as well as analyses conducted using data from healthy volunteers and from paediatric malaria patients. Specifically, Tan *et al *[47] modelled the PK of AS and DHA simultaneously utilizing extensive sampling data from 91 healthy Korean adults administered oral AS. The data were fit to a parent-metabolite model with first-order AS absorption, a one-compartment model for AS and a two-compartment model for DHA. Adjusting for the median weight of the study population (61.5 kg), the final estimates for AS CL/F and V/F were 19 L/kg/hr and 20 L/kg, respectively. Similarly adjusting for median weight, DHA central clearance and central volume of distribution were 1.52 L/kg/hr and 1.58 L/kg, respectively, with weight as a statistically significant covariate on DHA apparent clearance. The only other significant covariate-parameter relationship identified in the model was the effect of food intake on the AS absorption rate constant, with a reduction in absorption rate of 84% associated with administration of AS with a high fat, high calorie meal. Inter-individual variability was estimated on five of the modelled parameters, with the highest inter-individual variability observed for Ka (%CV = 112%) and AS V/F (%CV = 57.4%).

Stepniewska *et al *[48] described the PopPK of DHA following oral AS administration in children (6 months - 5 years) with uncomplicated falciparum malaria. AS and DHA pharmacokinetic data were obtained from 70 children who received AS and amodiaquine, but only DHA data could be modelled. Samples were collected once in the first dosing interval and once in the third dosing interval. The authors modelled DHA data using a one-compartment model with first-order input. They estimated DHA CL/F as 0.636 L/kg/hr for the first dosing period, with a substantial additive increase of 0.760 L/kg/hr being associated with the third dosing period. The authors speculated that this modelled increase in clearance reflected pharmacokinetic changes related to resolution of acute illness. DHA apparent volume of distribution, which was not modelled as varying between dosing periods, was estimated as 2.285 L/kg, with age modelled as a covariate on volume. The authors noted that either age or weight explained a significant portion of the variability on volume, but that the two covariates were not independent. Inter-individual variability was modelled on DHA apparent volume of distribution (%CV = 47%), but no other parameter.

### Rectal administration: AS and DHA pharmacokinetics

The bioavailability of rectally administered AS, as assessed by exposure to DHA, in paediatric patients with moderately severe malaria was estimated to be 23% in patients administered a dose of 20 mg/kg and 58% in patients administered 10 mg/kg [15]. In one rectal-oral crossover study in healthy volunteers, the mean bioavailability of rectal AS relative to oral AS, as assessed by exposure to DHA, was 54.9% [42]. However, in a study of similar design, no statistically significant differences in DHA AUC_0-t _following oral and rectal AS administration were observed, although AS AUC was significantly larger and DHA Cmax significantly smaller following rectal, as compared to oral, administration [30]. The inconsistent findings of these two studies may relate to the difficulty of defining a sampling schedule able to optimally capture the unique concentration-time profiles associated with different routes of administration.

Tables 9 and 10[15,30,42,49,50] summarize the PK findings of the identified rectal AS administration studies. Tmax for AS following rectal administration occurred on average between 0.58 - 1.43 hours. AS half-life was estimated in only two studies, with half-life estimates of 0.9 - 0.95 hours. These longer half-life estimates may reflect absorption rate-limited elimination of AS. Following rectal administration of AS, DHA concentrations peaked between 1.13 - 2.0 hours, and DHA was eliminated with a half-life averaging 0.79 - 1.8 hours. Only one non-PopPK study [15] reported estimates of DHA apparent clearance and volume following rectal AS; those values were 2.6 - 3.9 L/kg/hr and 4.4 - 5.9 L/kg, respectively. As would be expected given that rectal AS administration avoids first-pass metabolism, the discrepancy in AS and DHA AUC values is not as striking with rectal, as compared with oral, administration of AS.

**Table 9:** Summary of AS pharmacokinetic results following rectal AS administration.

**Ref**.	Subjects & Regimen	Cmax (ng/mL)	Tmax (hours)	Half-life (hours)	AUC (ng*hr/mL)
[49] Sirivichay *et al*, 2007	16 paediatric patients with uncomplicated falciparum malaria 10 mg/kg (n = 7) or 20 mg/kg (n = 9) AS as rectal suppositories	507; 561†^a^	0.8; 1.0^a^	0.9; 0.9^a^	692; 1076†^a^

[50] Halpaap *et al*, 1998	12 paediatric patients with uncomplicated falciparum malaria 50 mg AS as rectal suppository [0.86 - 2.55 mg/kg AS]	90†	0.58		

[30] Navaratnam *et al*, 1998	12 healthy Malaysian adults 200 mg AS as rectal suppository	448.5	1.43	0.95	796

Estimates from PopPK studies are described in the text. Values given as mean unless otherwise specified.

†Units converted to uniform scale a. Median

**Table 10:** Summary of DHA pharmacokinetic results following rectal AS administration.

**Ref**.	Subjects & Regimen	Cmax (ng/mL)	Tmax (hours)	Clearance (L/kg/hr)	Volume (L/kg)	Half-life (hours)	AUC (ng*hr/mL)
[49] Sirivichay *et al*, 2007	16 paediatric patients with uncomplicated falciparum malaria 10 mg/kg (n = 7) or 20 mg/kg (n = 9) AS as rectal suppositories	898; 1535†^a^	1.5; 2.0^a^			1.3; 1.8^a^	2403; 5633†^a^

[50] Halpaap *et al*, 1998	12 paediatric patients with uncomplicated falciparum malaria 50 mg AS as rectal suppository [0.86 - 2.55 mg/kg AS]	180†	1.13				

[42] Awad *et al*, 2004	12 healthy Sudanese adults 200 mg AS as rectal suppository	219.1†	1.95			1.21	1185.17

[30] Navaratnam *et al*, 1998	12 healthy Malaysian adults 200 mg AS as rectal suppository	385.6†	1.80				AUC_0-t_ 965

Krishna *et al*, 2001 [15]	34 Ghanaian children (8 months - 7 years) with moderate falciparum malaria Group 1: AS 10 mg/kg as rectal suppository, IV AS 2.4 mg/kg 12 hr later Group 2: AS 20 mg/kg AS as rectal suppository, IV AS 2.4 mg/kg 12 hr later Group 3: 2.4 mg/kg IV AS, 20 mg/kg AS as rectal suppository 12 hr later	Group 1: 682†^a^ Group 2 & 3: 881†^a^	Group 1: 1.7^a^ Tlag: 0.63^a^ Group 2 & 3: 1.8^a^ Tlag: 0.37^a^	Group 1: 2.6 Group 2 & 3: 3.9	Group 1: 4.4^a^ Group 2 & 3: 5.9^a^	Group 1: 0.79^a^ Group 2 & 3: 0.85^a^	Group 1: 2787 †^a^ Group 2 & 3: 3753†^a^

Estimates from PopPK studies are described in the text. Values given as mean unless otherwise specified

†Units converted to uniform scale a. Median

### Rectal administration: population pharmacokinetic analyses

Two population pharmacokinetic analyses of data obtained following rectal AS administration were identified. Simpson *et al *[51] described the population pharmacokinetics of DHA following rectal AS administration to adult and paediatric patients with moderately severe falciparum malaria. Patients were administered a single dose of 10 mg/kg AS with follow-up treatment administered orally. AS concentrations could not be successfully modelled. DHA concentrations (424 levels) obtained from 164 patients were fit to a one-compartment model with fixed, lagged, first-order input (DHA appearance rate: 0.2/hr; lag: 0.14 hr). Gender and weight were identified as important covariates in the model, with DHA CL/F of 3.17 L/kg/hr for males and 2.03 L/kg/hr for females. DHA V/F was estimated as increasing from 1.81 L/kg for a 15 kg subject to 6.34 L/kg for a 70 kg subject. Estimated inter-individual variability was 62% for CL/F and 75% for V/F.

Karunajeewa *et al *[52] conducted population pharmacokinetic analysis of AS and DHA data following administration of 10 - 15 mg/kg rectal AS (2 doses, 12 hours apart) to 47 paediatric uncomplicated falciparum or vivax malaria patients in Papua New Guinea. AS data and DHA data were each fit to a one-compartment model; first-order AS absorption was modelled. Due to identifiability concerns, the volume of distribution estimates for AS and DHA were set equal (41.8 L). Weight was an influential covariate on volume. The AS CL/F (mean ± SD) was determined to be 121.2 ± 35.4 L/hr and DHA CL/F to be 44.9 ± 13.0 L/hr. Average AS and DHA half-life estimates were 0.27 and 0.71 hours, respectively. The absorption half-life was estimated as 2.3 hours. The model included a bioavailability term for the second dose relative to the first dose (72%). The authors conjectured that higher core body temperature when the first dose was administered may have resulted in enhanced rectal blood flow and, therefore, absorption.

### Artesunate and DHA pharmacokinetics in paediatric patients

The two previously described population pharmacokinetic models describing AS/DHA PK following rectal AS administration were conducted using data from a mixed adult and paediatric population [51] or an exclusively paediatric population [52]; in both of these analyses, weight represented a significant covariate on DHA apparent volume of distribution. In the PopPK analysis of DHA following oral AS administration to young children, Stepniewska *et al *[48], determined that either weight or age could explain a significant portion of the between subject variability in DHA volume of distribution. These findings suggest that weight, or a highly correlated covariates such as age, is an important predictor variable for DHA PK in paediatric patients. The practice of utilizing AS regimens targeted to a mg/kg dosage range should somewhat aid in minimizing weight-based variability in exposure. However, further study would be required in the paediatric population to assess if patient age is an important source of variability beyond that explained by body weight alone. Such study would optimally focus on infants and very young children since the most marked differences in drug metabolism and other physiologic processes would be expected in this patient population.

### Artesunate and DHA pharmacokinetics in pregnant women

Two pharmacokinetic trials have been conducted to characterize AS/DHA pharmacokinetic changes that may be associated with the physiologic changes of pregnancy. McGready *et al *[43] modelled the PK of DHA following oral administration of AS to 2^nd ^and 3^rd ^trimester pregnant women with acute uncomplicated malaria. Population modelling yielded DHA CL/F and V/F estimates of 1.77 L/kg/hr and 4.63 L/kg. Non-compartmental analysis of their data yielded estimates of 4.0 L/kg/hr for CL/F and 3.4 L/kg for V/F. The authors noted that exposure to DHA following oral AS administration to the pregnant women in the study was substantially lower than that observed in non-pregnant subjects in previous studies.

Onyamboko *et al *[53] examined the PK of DHA following the oral administration of 200 mg AS to 26 2^nd ^and 3^rd ^trimester pregnant women with asymptomatic falciparum parasitaemia, the same women 3 months post-partum, and 25 matched asymptomatic parasitaemic female controls in the Democratic Republic of Congo. The median DHA CL/F was 1.39 L/kg/hr, 1.26 L/kg/hr, and 1.07 L/kg/hr for pregnant, post-partum, and non-pregnant control subjects, respectively. Median DHA V/F was 2.84 L/kg for pregnant, 3.00 L/kg for post-partum, and 2.45 L/kg for non-pregnant control subjects. DHA AUC was significantly different (geometric mean ratio: 0.68, 90% CI: 0.57 - 0.81) for the pregnant as compared to control subjects; however, DHA AUC values for pregnant women and the same women at three months post-partum were relatively similar. A population pharmacokinetic analysis [54] of the AS and DHA data from pregnant and control women in the Onyamboko *et al *study modelled the data using mixed-order absorption with a one-compartment model for AS and a one-compartment model for DHA; in that analysis, pregnancy was associated with a significant increase in DHA CL/F, as well as a trend towards increased volume of distribution.

### Artemisinin resistance in *Plasmodium falciparum *malaria

The recent emergence in western Cambodia of *P. falciparum *with reduced susceptibility to artemisinin derivatives has been the source of substantial concern; questions regarding the relationship between the pharmacokinetics of these derivatives and the observed delayed parasite clearance times have been posed. Dondorp *et al *[37] assessed the efficacy of two regimens of AS for uncomplicated falciparum malaria at a site in western Cambodia, where reduced susceptibility was expected, and another in northwestern Thailand, where substantially reduced susceptibility was not anticipated. These regimens consisted of 2 mg/kg/day oral AS monotherapy × 7 days or 4 mg/kg/day oral AS × 3 days followed by two doses of mefloquine. As expected, patients in Cambodia displayed significantly longer parasite clearance times as compared to patients in Thailand. However, no apparent clinically relevant differences in AS and DHA pharmacokinetics were observed between the two study sites. Additionally, no relationship between measures of AS or DHA exposure and parasite clearance time was observed. These results suggest that the observed reduced artemisinin susceptibility of *P. falciparum *in western Cambodia is not highly sensitive to PK parameters for AS and DHA within the 2 - 4 mg/kg/day AS dosage range.

### Effect of infection status on artesunate and DHA pharmacokinetics

Multiple studies have attempted to investigate and characterize any changes in AS and DHA PK associated with malaria infection. Two of the studies described above, conducted by Stepniewska *et al *[48] and Karbwang *et al *[31], determined that the PK of orally administered AS may differ in the acute stage of infection as compared to the convalescent stage. Stepniewska *et al *[48] determined that DHA clearance was substantially lower on the first day of treatment as compared to the third day. Karbwang *et al *[31] determined that DHA Cmax was significantly decreased, and AUC not significantly changed, on the first day of treatment as compared to the fifth day. On the first day of treatment, higher AS clearance was also reported. Newton *et al *[19] used bioassay data to investigate the anti-malarial activity in patients with falciparum malaria during the patients' acute and convalescent phases. The analysis indicated that anti-malarial activity AUC and Cmax were two-fold higher in the acute phase as compared to the convalescent phase for subjects administered the same dose of oral AS. Correspondingly, apparent clearance and volume of distribution of anti-malarial activity were significantly smaller in the acute phase of infection. Although these three studies do not fully align regarding the effect of disease resolution on AS/DHA PK, perhaps due to the use of differing populations, sampling time points, and time course of sampling, taken together these studies do suggest that some alteration in PK may occur over the course of treatment. It should be noted that changes over the course of treatment are likely not due to time-dependency of AS or DHA PK, as has been observed with various other artemisinin derivatives; following oral administration of AS over a typical treatment course, time-dependent kinetics are not apparent [55].

A direct comparison of healthy and parasitaemic subjects was conducted by Teja-Isavadharm *et al *[35], who studied the PK of DHA following oral AS administration to six healthy adults and six adult falciparum malaria patients. The investigators determined that AUC and Cmax of DHA were significantly higher in subjects with malaria as compared to healthy subjects. Binh *et al *[9] obtained similar results when comparing the PK in eight patients with falciparum malaria and ten healthy subjects. However, given the relatively small size of both the Binh *et al *[9] and Teja-Isavadharm *et al *[35] studies, drawing definitive conclusions regarding differences in PK between healthy and infected subjects is not possible at present. Nonetheless, as DHA clearance is dependent upon hepatic blood flow, a reduction in clearance, and consequently an increase in exposure, associated with acute infection would be consistent with DHA's known pharmacokinetic properties.

### Drug-drug interactions

Given the metabolic pathways of AS (esterase-catalyzed hydrolysis) and DHA (UGT-mediated conjugation), AS should not be susceptible to the many common drug-drug interactions involving CYP450 enzymes. Agents evaluated for their drug interaction potential with orally administered AS include atovaquone-proguanil [56], sulphadoxine-pyrimethamine [57], pyronaridine [47], mefloquine [24], chlorproguanil-dapsone [34], artemisinin [41], and amodiaquine [27]. AS coadministration does not appear to alter the PK of atovaquone-proguanil [56] or sulphadoxine-pyrimethamine [57]. No significant change in DHA AUC was detected when AS was coadministered with mefloquine [24]. In the PopPK analysis of AS and DHA PK following oral AS administration by Tan *et *al (described above), coadministration of AS with the Mannich-base derivative pyronaridine was not determined to exert a significant influence on AS or DHA pharmacokinetics [47]. Multiple dose administration of AS did not alter the PK of artemisinin; however, artemisinin coadministration with AS in ten healthy adults was associated with a more than two-fold increase in DHA AUC, a finding which led the authors to speculate that artemisinin may act as a UGT inhibitor [41]. Finally, AS coadministration with chlorproguanil-dapsone did not produce significant alterations in chlorproguanil or dapsone PK, although moderate increases in exposure to the metabolites chlorcycloguanil and monoacetyl dapsone were detected. No clinically significant alterations of AS and DHA pharmacokinetics were found to be associated with AS-chlorproguanil-dapsone combination therapy [34].

Orrell *et al *[27] investigated the drug interaction potential of artesunate and amodiaquine. The authors conducted a crossover study in which 12 healthy African adults received 4 mg/kg AS on day 0 and either amodiaquine or amodiaquine+AS on day 7, with the alternative regimen administered on day 28. The investigators determined that when amodiaquine and AS were coadministered, the mean DHA AUC was approximately 33% lower, the mean DHA Cmax was 49% lower, and the mean DHA half-life was 57% longer than when AS was administered alone [27]. The AUC of the amodiaquine metabolite desethylamodiaquine was determined to be 45% lower when amodiaquine was coadministered with AS. However, the subject with the highest desethylamodiaquine AUC during amodiaquine+AS coadministration was excluded from the amodiaquine drug interaction analysis [27]. Orrell *et al *do not speculate on the source of the interaction. Given the small size of the study, and the lack of any clear physiologic basis for the observed interaction, further study would be needed to fully characterize this potential drug-drug interaction.

## Conclusion

AS is a clinically versatile artemisinin derivative utilized for the treatment of mild to severe malaria infection. Given the therapeutic significance of AS, and the necessity of appropriate AS dosing, substantial research has been performed investigating the pharmacokinetics of AS and its active metabolite DHA. The results of the studies identified in this review indicate that administration of IV AS produces an AS Cmax of substantially greater magnitude than observed with any other route of administration. Following IV administration, AS hydrolysis to DHA occurs rapidly, producing DHA peak concentrations within 25 minutes post-dose. AS and DHA display average clearance values of 2 - 3 L/kg/hr and 0.5 - 1.5 L/kg/hr, respectively, with volume estimates averaging 0.1 - 0.3 L/kg for AS and 0.5 - 1.0 L/kg for DHA. IM administration of AS is associated with high bioavailability, as assessed by DHA exposure. Although generally displaying similar PK to IV AS, IM AS does produce lower Cmax, higher V/F, and longer half-life values for AS, as well as longer Tmax values for DHA, than IV administration.

Following oral AS administration, peak AS concentrations are attained within an hour, with AS eliminated with a half-life of 20 - 45 minutes. DHA Cmax values occur within two hours post-dose; DHA half-life values average 0.5 - 1.5 hours. A marked discrepancy in AS and DHA AUC values is apparent following oral AS administration, with DHA AUC values commonly determined to be more than 10-fold higher than corresponding AS AUC values. The PK parameters obtained in studies with rectal AS administration are generally similar to those obtained in studies with oral administration, although AS Tmax is delayed and AS half-life extended. PopPK analyses of AS/DHA data following oral and rectal AS administration suggest that weight and pregnancy represent influential predictors of DHA pharmacokinetics following AS administration.

To date, drug interactions studies of AS with various other anti-malarial agents have not yielded strong evidence of clinically relevant drug-drug interactions involving AS. Several relatively small studies examining the effects of infection on AS and DHA PK indicate that acute malaria infection may be associated with PK changes; however, determining the exact nature of such changes will require further study. Similarly, present evidence suggests that pregnancy may result in PK changes which will require further study for full elucidation.

## Competing interests

Chang-Sik Shin is an employee of Shin Poong Pharmaceuticals.

## Authors' contributions

CAM, SD, IBF, DJ, CS and LF all made substantial contributions to the conception, organization, and revision of the review. All of the authors critically reviewed the manuscript and approved the final version for submission.

## References

[B1] World Health Organization Guidelines for the treatment of malaria 2011 2 Geneva, Switzerland: World Health Organization Rev. 1

[B2] Lindegardh N Hanpithakpong W Kamanikom B Singhasivanon P Socheat D Yi P Dondorp AM McGready R Nosten F White NJ Day NP Major pitfalls in the measurement of artemisinin derivatives in plasma in clinical studies J Chromatogr B Analyt Technol Biomed Life Sci 2008 876 54 60 10.1016/j.jchromb.2008.10.021 18980865

[B3] Li Q Xie LH Haeberle A Zhang J Weina P The evaluation of radiolabeled artesunate on tissue distribution in rats and protein binding in humans Am J Trop Med Hyg 2006 75 817 826 17123971

[B4] Xie LH Li Q Zhang J Weina PJ Pharmacokinetics, tissue distribution and mass balance of radiolabeled dihydroartemisinin in male rats Malar J 2009 8 112 10.1186/1475-2875-8-112 19470172 PMC2694832

[B5] Batty KT Ilett KF Davis TM Protein binding and alpha: beta anomer ratio of dihydroartemisinin in vivo Br J Clin Pharmacol 2004 57 529 533 10.1046/j.1365-2125.2003.02045.x 15025754 PMC1884468

[B6] Batty KT Iletr KE Powell SM Martin J Davis TM Relative bioavailability of artesunate and dihydroartemisinin: investigations in the isolated perfused rat liver and in healthy Caucasian volunteers Am J Trop Med Hyg 2002 66 130 136 12135281 10.4269/ajtmh.2002.66.130

[B7] Batty KT Le AT Ilett KF Nguyen PT Powell SM Nguyen CH Truong XM Vuong VC Huynh VT Tran QB Nguyen VM Davis TM A pharmacokinetic and pharmacodynamic study of artesunate for vivax malaria Am J Trop Med Hyg 1998 59 823 827 9840605 10.4269/ajtmh.1998.59.823

[B8] Batty KT Thu LT Davis TM Ilett KF Mai TX Hung NC Tien NP Powell SM Thien HV Binh TQ Kim NV A pharmacokinetic and pharmacodynamic study of intravenous vs oral artesunate in uncomplicated falciparum malaria Br J Clin Pharmacol 1998 45 123 129 9491824 10.1046/j.1365-2125.1998.00655.x PMC1873351

[B9] Binh TQ Ilett KF Batty KT Davis TM Hung NC Powell SM Thu LT Thien HV Phuong HL Phuong VD Oral bioavailability of dihydroartemisinin in Vietnamese volunteers and in patients with falciparum malaria Br J Clin Pharmacol 2001 51 541 546 10.1046/j.1365-2125.2001.01395.x 11422013 PMC2014487

[B10] Ilett KF Batty KT Powell SM Binh TQ Thu le TA Phuong HL Hung NC Davis TM The pharmacokinetic properties of intramuscular artesunate and rectal dihydroartemisinin in uncomplicated falciparum malaria Br J Clin Pharmacol 2002 53 23 30 10.1046/j.0306-5251.2001.01519.x 11849191 PMC1874553

[B11] Li Q Cantilena LR Leary KJ Saviolakis GA Miller RS Melendez V Weina PJ Pharmacokinetic profiles of artesunate after single intravenous doses at 0.5, 1, 2, 4, and 8 mg/kg in healthy volunteers: a phase I study Am J Trop Med Hyg 2009 81 615 621 10.4269/ajtmh.2009.09-0150 19815876

[B12] Nealon C Dzeing A Muller-Romer U Planche T Sinou V Kombila M Kremsner PG Parzy D Krishna S Intramuscular bioavailability and clinical efficacy of artesunate in gabonese children with severe malaria Antimicrob Agents Chemother 2002 46 3933 3939 10.1128/AAC.46.12.3933-3939.2002 12435698 PMC132755

[B13] Newton PN Barnes KI Smith PJ Evans AC Chierakul W Ruangveerayuth R White NJ The pharmacokinetics of intravenous artesunate in adults with severe falciparum malaria Eur J Clin Pharmacol 2006 62 1003 1009 10.1007/s00228-006-0203-2 17089111

[B14] Davis TM Phuong HL Ilett KF Hung NC Batty KT Phuong VD Powell SM Thien HV Binh TQ Pharmacokinetics and pharmacodynamics of intravenous artesunate in severe falciparum malaria Antimicrob Agents Chemother 2001 45 181 186 10.1128/AAC.45.1.181-186.2001 11120963 PMC90258

[B15] Krishna S Planche T Agbenyega T Woodrow C Agranoff D Bedu-Addo G Owusu-Ofori AK Appiah JA Ramanathan S Mansor SM Navaratnam V Bioavailability and preliminary clinical efficacy of intrarectal artesunate in Ghanaian children with moderate malaria Antimicrob Agents Chemother 2001 45 509 516 10.1128/AAC.45.2.509-516.2001 11158748 PMC90320

[B16] Hess KM Goad JA Arguin PM Intravenous artesunate for the treatment of severe malaria Ann Pharmacother 2010 44 1250 1258 10.1345/aph.1M732 20551300

[B17] Gautam A Ahmed T Batra V Paliwal J Pharmacokinetics and pharmacodynamics of endoperoxide antimalarials Curr Drug Metab 2009 10 289 306 10.2174/138920009787846323 19442090

[B18] Ilett KF Ethell BT Maggs JL Davis TM Batty KT Burchell B Binh TQ Thu le TA Hung NC Pirmohamed M Park BK Edwards G Glucuronidation of dihydroartemisinin in vivo and by human liver microsomes and expressed UDP-glucuronosyltransferases Drug Metab Dispos 2002 30 1005 1012 10.1124/dmd.30.9.1005 12167566

[B19] Newton P Suputtamongkol Y Teja-Isavadharm P Pukrittayakamee S Navaratnam V Bates I White N Antimalarial bioavailability and disposition of artesunate in acute falciparum malaria Antimicrob Agents Chemother 2000 44 972 977 10.1128/AAC.44.4.972-977.2000 10722499 PMC89800

[B20] Ittarat W Looareesuwan S Pootrakul P Sumpunsirikul P Vattanavibool P Meshnick SR Effects of alpha-thalassemia on pharmacokinetics of the antimalarial agent artesunate Antimicrob Agents Chemother 1998 42 2332 2335 9736558 10.1128/aac.42.9.2332 PMC105828

[B21] Hien TT Davis TM Chuong LV Ilett KF Sinh DX Phu NH Agus C Chiswell GM White NJ Farrar J Comparative pharmacokinetics of intramuscular artesunate and artemether in patients with severe falciparum malaria Antimicrob Agents Chemother 2004 48 4234 4239 10.1128/AAC.48.11.4234-4239.2004 15504846 PMC525450

[B22] Olliaro PL Nair NK Sathasivam K Mansor SM Navaratnam V Pharmacokinetics of artesunate after single oral administration to rats BMC Pharmacol 2001 1 12 10.1186/1471-2210-1-12 11835690 PMC65525

[B23] Chanthap L Tsuyuoka R Na-Bangchang K Nivanna N Suksom D Sovannarith T Socheat D Investigation of bioavailability, pharmacokinetics and safety of new pediatric formulations of artesunate and mefloquine Southeast Asian J Trop Med Public Health 2005 36 34 43 15906639

[B24] Davis TM England M Dunlop AM Page-Sharp M Cambon N Keller TG Heidecker JL Ilett KF Assessment of the effect of mefloquine on artesunate pharmacokinetics in healthy male volunteers Antimicrob Agents Chemother 2007 51 1099 1101 10.1128/AAC.01253-06 17178798 PMC1803145

[B25] Benakis A Paris M Loutan L Plessas CT Plessas ST Pharmacokinetics of artemisinin and artesunate after oral administration in healthy volunteers Am J Trop Med Hyg 1997 56 17 23 9063354 10.4269/ajtmh.1997.56.17

[B26] Navaratnam V Ramanathan S Wahab MS Siew Hua G Mansor SM Kiechel JR Vaillant M Taylor WR Olliaro P Tolerability and pharmacokinetics of non-fixed and fixed combinations of artesunate and amodiaquine in Malaysian healthy normal volunteers Eur J Clin Pharmacol 2009 65 809 821 10.1007/s00228-009-0656-1 19404632 PMC2714898

[B27] Orrell C Little F Smith P Folb P Taylor W Olliaro P Barnes KI Pharmacokinetics and tolerability of artesunate and amodiaquine alone and in combination in healthy volunteers Eur J Clin Pharmacol 2008 64 683 690 10.1007/s00228-007-0452-8 18415093 PMC2426925

[B28] Na-Bangchang K Karbwang J Congpoung K Thanavibul A Ubalee R Pharmacokinetic and bioequivalence evaluation of two generic formulations of oral artesunate Eur J Clin Pharmacol 1998 53 375 376 10.1007/s002280050397 9516041

[B29] Diem Thuy LT Ngoc Hung L Danh PT Na-Bangchang K Absence of time-dependent artesunate pharmacokinetics in healthy subjects during 5-day oral administration Eur J Clin Pharmacol 2008 64 993 998 10.1007/s00228-008-0506-6 18600318

[B30] Navaratnam V Mansor SM Mordi MN Akbar A Abdullah MN Comparative pharmacokinetic study of oral and rectal formulations of artesunic acid in healthy volunteers Eur J Clin Pharmacol 1998 54 411 414 10.1007/s002280050484 9754985

[B31] Karbwang J Na-Bangchang K Congpoung K Thanavibul A Harinasuta T Pharmacokinetics of oral artesunate in thai patients with uncomplicated falciparum malaria Clin Drug Investig 1998 15 37 43 10.2165/00044011-199815010-00005 18370464

[B32] Krudsood S Looareesuwan S Tangpukdee N Wilairatana P Phumratanaprapin W Leowattana W Chalermrut K Ramanathan S Navaratnam V Olliaro P Vaillant M Kiechel JR Taylor WR New fixed-dose artesunate-mefloquine formulation against multidrug-resistant *Plasmodium falciparum *in adults: a comparative phase IIb safety and pharmacokinetic study with standard-dose nonfixed artesunate plus mefloquine Antimicrob Agents Chemother 2010 54 3730 3737 10.1128/AAC.01187-09 20547795 PMC2935027

[B33] Sinou V Malaika LT Taudon N Lwango R Alegre SS Bertaux L Sugnaux F Parzy D Benakis A Pharmacokinetics and pharmacodynamics of a new ACT formulation: Artesunate/Amodiaquine (TRIMALACT) following oral administration in African malaria patients Eur J Drug Metab Pharmacokinet 2009 34 133 142 10.1007/BF03191163 20166428

[B34] Miller AK Bandyopadhyay N Wootton DG Duparc S Kirby PL Winstanley PA Ward SA Pharmacokinetics of chlorproguanil, dapsone, artesunate and their major metabolites in patients during treatment of acute uncomplicated *Plasmodium falciparum *malaria Eur J Clin Pharmacol 2009 65 977 987 10.1007/s00228-009-0672-1 19517101

[B35] Teja-Isavadharm P Watt G Eamsila C Jongsakul K Li Q Keeratithakul G Sirisopana N Luesutthiviboon L Brewer TG Kyle DE Comparative pharmacokinetics and effect kinetics of orally administered artesunate in healthy volunteers and patients with uncomplicated falciparum malaria Am J Trop Med Hyg 2001 65 717 721 11791963 10.4269/ajtmh.2001.65.717

[B36] Ramharter M Kurth F Schreier AC Nemeth J Glasenapp I Belard S Schlie M Kammer J Koumba PK Cisse B Mordmuller B Lell B Issifou S Oeuvray C Fleckenstein L Kremsner PG Fixed-dose pyronaridine-artesunate combination for treatment of uncomplicated falciparum malaria in pediatric patients in Gabon J Infect Dis 2008 198 911 919 10.1086/591096 18694333

[B37] Dondorp AM Nosten F Yi P Das D Phyo AP Tarning J Lwin KM Ariey F Hanpithakpong W Lee SJ Ringwald P Silamut K Imwong M Chotivanich K Lim P Herdman T An SS Yeung S Singhasivanon P Day NP Lindegardh N Socheat D White NJ Artemisinin resistance in *Plasmodium falciparum *malaria N Engl J Med 2009 361 455 467 10.1056/NEJMoa0808859 19641202 PMC3495232

[B38] Ramharter M Kurth FM Belard S Bouyou-Akotet MK Mamfoumbi MM Agnandji ST Missinou MA Adegnika AA Issifou S Cambon N Heidecker JL Kombila M Kremsner PG Pharmacokinetics of two paediatric artesunate mefloquine drug formulations in the treatment of uncomplicated falciparum malaria in Gabon J Antimicrob Chemother 2007 60 1091 1096 10.1093/jac/dkm355 17884832

[B39] Mwesigwa J Parikh S McGee B German P Drysdale T Kalyango JN Clark TD Dorsey G Lindegardh N Annerberg A Rosenthal PJ Kamya MR Aweeka F Pharmacokinetics of artemether-lumefantrine and artesunate-amodiaquine in children in Kampala, Uganda Antimicrob Agents Chemother 2010 54 52 59 10.1128/AAC.00679-09 19841149 PMC2798532

[B40] Na-Bangchang K Krudsood S Silachamroon U Molunto P Tasanor O Chalermrut K Tangpukdee N Matangkasombut O Kano S Looareesuwan S The pharmacokinetics of oral dihydroartemisinin and artesunate in healthy Thai volunteers Southeast Asian J Trop Med Public Health 2004 35 575 582 15689069

[B41] Zhang SQ Hai TN Ilett KF Huong DX Davis TM Ashton M Multiple dose study of interactions between artesunate and artemisinin in healthy volunteers Br J Clin Pharmacol 2001 52 377 385 10.1046/j.0306-5251.2001.01461.x 11678781 PMC2014589

[B42] Awad MI Eltayeb IB Baraka OZ Behrens RH Alkadru AM Pharmacokinetics of artesunate following oral and rectal administration in healthy Sudanese volunteers Trop Doct 2004 34 132 135 15267037 10.1177/004947550403400302

[B43] McGready R Stepniewska K Ward SA Cho T Gilveray G Looareesuwan S White NJ Nosten F Pharmacokinetics of dihydroartemisinin following oral artesunate treatment of pregnant women with acute uncomplicated falciparum malaria Eur J Clin Pharmacol 2006 62 367 371 10.1007/s00228-006-0118-y 16552504

[B44] Teja-Isavadharm P Watt G Eamsila C Jongsakul K Li Q Keeratithakul G Sirisopana N Luesutthiviboon L Brewer TG Kyle DE Comparative pharmacokinetics and effect kinetics of orally administered artesunate in healthy volunteers and patients with uncomplicated falciparum malaria Am J Trop Med Hyg 2001 65 717 721 11791963 10.4269/ajtmh.2001.65.717

[B45] Newton PN van Vugt M Teja-Isavadharm P Siriyanonda D Rasameesoroj M Teerapong P Ruangveerayuth R Slight T Nosten F Suputtamongkol Y Looareesuwan S White NJ Comparison of oral artesunate and dihydroartemisinin antimalarial bioavailabilities in acute falciparum malaria Antimicrob Agents Chemother 2002 46 1125 1127 10.1128/AAC.46.4.1125-1127.2002 11897605 PMC127101

[B46] Bethell DB Teja-Isavadharm P Cao XT Pham TT Ta TT Tran TN Nguyen TT Pham TP Kyle D Day NP White NJ Pharmacokinetics of oral artesunate in children with moderately severe *Plasmodium falciparum *malaria Trans R Soc Trop Med Hyg 1997 91 195 198 10.1016/S0035-9203(97)90222-4 9196768

[B47] Tan B Naik H Jang IJ Yu KS Kirsch LE Shin CS Craft JC Fleckenstein L Population pharmacokinetics of artesunate and dihydroartemisinin following single- and multiple-dosing of oral artesunate in healthy subjects Malar J 2009 8 304 10.1186/1475-2875-8-304 20021657 PMC2806381

[B48] Stepniewska K Taylor W Sirima SB Ouedraogo EB Ouedraogo A Gansane A Simpson JA Morgan CC White NJ Kiechel JR Population pharmacokinetics of artesunate and amodiaquine in African children Malar J 2009 8 200 10.1186/1475-2875-8-200 19691851 PMC3224946

[B49] Sirivichayakul C Sabchareon A Pengsaa K Thaiarporn I Chaivisuth A Na-Bangchang K Wisetsing P Chanthavanich P Pojjaroen-Anant C Comparative study of the effectiveness and pharmacokinetics of two rectal artesunate/oral mefloquine combination regimens for the treatment of uncomplicated childhood falciparum malaria Ann Trop Paediatr 2007 27 17 24 10.1179/146532807X170466 17469728

[B50] Halpaap B Ndjave M Paris M Benakis A Kremsner PG Plasma levels of artesunate and dihydroartemisinin in children with *Plasmodium falciparum *malaria in Gabon after administration of 50-milligram artesunate suppositories Am J Trop Med Hyg 1998 58 365 368 9546420 10.4269/ajtmh.1998.58.365

[B51] Simpson JA Agbenyega T Barnes KI Di Perri G Folb P Gomes M Krishna S Krudsood S Looareesuwan S Mansor S McIlleron H Miller R Molyneux M Mwenechanya J Navaratnam V Nosten F Olliaro P Pang L Ribeiro I Tembo M van Vugt M Ward S Weerasuriya K Win K White NJ Population pharmacokinetics of artesunate and dihydroartemisinin following intra-rectal dosing of artesunate in malaria patients PLoS Med 2006 3 e444 10.1371/journal.pmed.0030444 17132053 PMC1664603

[B52] Karunajeewa HA Ilett KF Dufall K Kemiki A Bockarie M Alpers MP Barrett PH Vicini P Davis TM Disposition of artesunate and dihydroartemisinin after administration of artesunate suppositories in children from Papua New Guinea with uncomplicated malaria Antimicrob Agents Chemother 2004 48 2966 2972 10.1128/AAC.48.8.2966-2972.2004 15273107 PMC478493

[B53] Onyamboko MA Meshnick SR Fleckenstein L Koch MA Atibu J Lokomba V Douoguih M Hemingway-Foday J Wesche D Ryder RW Bose C Wright LL Tshefu AK Capparelli EV Pharmacokinetics and pharmacodynamics of artesunate and dihydroartemisinin following oral treatment in pregnant women with asymptomatic *Plasmodium falciparum *infections in Kinshasa DRC Malar J 2011 10 49 10.1186/1475-2875-10-49 21352601 PMC3056842

[B54] Morris CA Onyamboko MA Capparelli E Koch MA Atibu J Lokomba V Douoguih M Hemingway-Foday J Wesche D Ryder RW Bose C Wright L Tshefu AK Meshnick S Fleckenstein L Population pharmacokinetics of artesunate and dihydroartemisinin in pregnant and non-pregnant women with malaria Malar J 2011 10 114 10.1186/1475-2875-10-114 21548983 PMC3098207

[B55] Diem Thuy LT Ngoc Hung L Danh PT Na-Bangchang K Absence of time-dependent artesunate pharmacokinetics in healthy subjects during 5-day oral administration Eur J Clin Pharmacol 2008 64 993 998 10.1007/s00228-008-0506-6 18600318

[B56] Van Vugt M Edstein MD Proux S Lay K Ooh M Looareesuwan S White NJ Nosten F Absence of an interaction between artesunate and atovaquone--proguanil Eur J Clin Pharmacol 1999 55 469 474 10.1007/s002280050658 10492061

[B57] Minzi OM Gupta A Haule AF Kagashe GA Massele AY Gustafsson LL Lack of impact of artesunate on the disposition kinetics of sulfadoxine/pyrimethamine when the two drugs are concomitantly administered Eur J Clin Pharmacol 2007 63 457 462 10.1007/s00228-007-0278-4 17333157

